# Real-World Clinical Outcomes of Transperineal Laser Ablation in BPH: A 12-Month Retrospective Analysis

**DOI:** 10.3390/jcm14176079

**Published:** 2025-08-28

**Authors:** Yusuf Özlülerden, Kürşat Küçüker, Sinan Çelen, Mesut Berkan Duran, Aykut Başer, Ahmet Baki Yagci, Ömer Levent Tuncay

**Affiliations:** 1 Department of Urology, Pamukkale University Faculty of Medicine, 20070 Denizli, Türkiye; yusufozlu35@hotmail.com (Y.Ö.); kursat_kucuker@hotmail.com (K.K.); sinancelen@hotmail.com (S.Ç.); drberkanduran@gmail.com (M.B.D.); omerleventtuncay@yahoo.com (Ö.L.T.); 2 Department of Urology, Faculty of Medicine, Bandırma Onyedi Eylül University, 10250 Bandırma, Türkiye; 3 Department of Radiology, Pamukkale University Faculty of Medicine, 20070 Denizli, Türkiye; byagci@pau.edu.tr

**Keywords:** benign prostatic hyperplasia, transperineal laser ablation, lower urinary tract symptoms, minimally invasive surgery, sexual function, prostate volume, ultrasound guidance

## Abstract

**Background/Objectives**: Benign prostatic hyperplasia (BPH) is a prevalent condition in aging men and a major cause of lower urinary tract symptoms (LUTSs). While traditional treatments such as transurethral resection of the prostate (TURP) are effective, they are associated with notable morbidity. Ultrasound-guided transperineal laser ablation (TPLA) has emerged as a minimally invasive alternative. This study aimed to assess the 12-month efficacy, safety, and functional outcomes of TPLA in patients with LUTS secondary to BPH. **Methods**: This was a single-center, retrospective observational cohort study including 53 patients with moderate-to-severe LUTS due to BPH who underwent TPLA between November 2021 and May 2024. Baseline and follow-up assessments were conducted at 1, 3, 6, and 12 months, including IPSS, Qmax, PVR, prostate volume (MRI), QoL, IIEF-5, and MSHQ-ED/Bother scores. The procedure was performed under local anesthesia using the EchoLaser™ system, and ablation was guided via real-time transrectal ultrasonography. **Results**: Statistically significant improvements were observed in IPSS (median decrease from 30 to 13), Qmax (5.5 to 13.0 mL/s), and PVR (200 to 85 mL). Prostate and adenoma volumes decreased by 41.2% and 58.3%, respectively. Quality of life scores improved, and erectile function remained stable. Ejaculatory function improved significantly based on MSHQ-ED and MSHQ-Bother scores. No major complications or conversions to surgery occurred. **Conclusions**: TPLA appears to be a safe, effective, and minimally invasive treatment modality for LUTS caused by BPH. It offers sustained symptomatic relief, prostate volume reduction, and preservation of sexual function, making it a promising alternative for patients unfit or unwilling to undergo invasive surgery.

## 1. Introduction

Benign prostatic hyperplasia (BPH) is a common condition in aging men and a major cause of lower urinary tract symptoms (LUTSs). The prevalence of BPH is approximately 70% in men aged 60–69 and increases up to 80% in those over 80 years of age [1,2]. As LUTS progress, patients often experience a significant decline in quality of life, which necessitates clinical intervention [2]. Although first-line treatment typically involves alpha-blockers and 5-alpha-reductase inhibitors, these pharmacological options may not provide sufficient efficacy or tolerability in all patients [3,4,5].

Transurethral resection of the prostate (TURP) is still regarded as the gold standard surgical approach. However, its invasive nature is associated with complications such as retrograde ejaculation, bleeding, infection, and urinary incontinence [6,7,8]. As a result, minimally invasive treatment options have garnered increasing attention in recent years.

Minimally invasive surgical techniques (MISTs) aim to offer similar efficacy to conventional surgical methods while reducing hospital stay and complication rates [9,10]. These include a variety of modalities such as Rezum, UroLift, prostatic artery embolization (PAE), Aquablation, and intraprostatic injections [11,12]. Among these, ultrasound-guided transperineal laser ablation (TPLA) has recently emerged as an innovative technique. TPLA involves the insertion of diode laser fibers into the prostate via a transperineal route, producing localized thermal coagulative necrosis [13,14]. This method stands out for its feasibility under local anesthesia or sedation, short procedure time, and favorable safety profile [11,15].

However, the long-term clinical outcomes of TPLA have yet to be fully established. Therefore, the aim of this retrospective study was to evaluate the efficacy and safety of TPLA in patients suffering from LUTS due to benign prostatic obstruction. Parameters such as procedure time, prostate and adenoma volume (assessed by MRI), Qmax, IPSS, and quality of life scores were analyzed at 1, 3, 6, and 12 months postoperatively. Through this, we aimed to contribute to the growing body of literature on the long-term clinical utility of TPLA.

## 2. Materials and Methods

### 2.1. Study Design and Participants

This single-center observational cohort study was conducted retrospectively between November 2021 and May 2024. A total of 53 male patients diagnosed with benign prostatic hyperplasia (BPH) and presenting with lower urinary tract symptoms (LUTS) were included. All patients underwent transperineal laser ablation (TPLA) and were followed for 12 months.

This study was conducted in accordance with the principles of the Declaration of Helsinki and was approved by the Pamukkale University Non-Interventional Clinical Research Ethics Committee (Approval No: 05, dated 14 March 2023).

### 2.2. Inclusion and Exclusion Criteria

Inclusion criteria were as follows: age ≥50 years; presence of moderate-to-severe lower urinary tract symptoms (LUTS) related to benign prostatic hyperplasia (BPH); documented prostate volume measured by transrectal ultrasonography (TRUS) or magnetic resonance imaging (MRI); a maximum urinary flow rate (Qmax) ≤15 mL/s; an International Prostate Symptom Score (IPSS) ≥13; and baseline post-void residual urine volume (PVR) measurement regardless of its presence or absence in non-catheterized patients. Patients with PVR >400 mL underwent further neuro-urological evaluation, including urodynamic studies, and were excluded if a neurogenic bladder was diagnosed; and no history of prior surgical treatment involving the prostate, urethra, or bladder.

Exclusion criteria included a history of surgery involving the prostate, bladder neck, or urethra; confirmed or suspected prostate or bladder cancer; abnormal prostate-specific antigen (PSA) levels (>4 ng/mL) without a prior negative biopsy; significant urethral stricture; active urinary tract infection; known neurological disorders affecting bladder function (e.g., multiple sclerosis, Parkinson’s disease, spinal cord injury); hypersensitivity to contrast agents; and patients with a median lobe hyperplasia of the prostate demonstrated by MRI and/or TRUS were excluded.

### 2.3. TPLA Procedure (EchoLaser™-Based Protocol)

Procedures were conducted under local anesthesia with Foley catheter placement prior to treatment. Catheter removal occurred on postoperative day 5 for non-catheterized patients and after two weeks of bladder training for chronic catheter users. In cases where patients developed acute urinary retention (glob vesicale) following catheter removal, re-catheterization was performed in accordance with the follow-up protocol. During subsequent visits, catheter removal was reattempted, and patients were monitored for the possible occurrence of acute urinary retention.

An EchoLaser™ system (SoracteLite™/EchoLaser X4, Elesta S.p.A., Calenzano, Florence, Italy), comprising a multi-source diode laser (1064 nm) and integrated planning software (Echolaser Smart Interface, ESI), was utilized with a biplanar TRUS probe (e.g., MyLab Eight eXP, Esaote, Genoa, Italy) for imaging [16,17,18,19,20]. Laser delivery was adjusted to a fixed starting power of approximately 5 W, reduced to 3.5 W (if necesseray) after about two minutes when vaporization and hyperechoic bubbles appeared on ultrasound. A standard energy dose of 3600 Joule was applied for prostates smaller than 50 mL, while 7200 Joule was delivered in cases where the prostate volume exceeded 50 mL.

Patients were positioned in lithotomy. One or two 21-gauge Chiba introducer needles per lobe were inserted percutaneously via a transperineal route under TRUS guidance. Optical fibers (300 µm quartz, flat-tip, Elesta S.p.A.) were introduced with a 10 mm protrusion beyond the needle tip. Fiber placement maintained ≥15 mm from the bladder neck and ≥10 mm from the prostatic capsule and urethra [16,18]. Fiber spacing of ~10 mm ensured optimal necrosis volume when multiple fibers were used. The pull-back technique (retracting the fiber by ca. 5–10 mm) was applied to extend ablation coverage in larger glands. Real-time TRUS imaging allowed verification and adjustment of fiber positioning and ablation effect throughout [16,21]. During and prior to the TPLA procedure, continuous cold saline irrigation (+4 °C) was applied to reduce the thermal effect of the laser energy.

### 2.4. Follow-Up and Evaluation Parameters

Patients were evaluated at baseline and at 1, 3, 6, and 12 months postoperatively. The following intraoperative parameters were recorded:

- Procedure duration (minutes);
- Pain intensity (VAS score);
- Total energy applied (Joules);
- Number of laser fibers used;

Variables assessed preoperatively and at follow-up visits included:;

- Serum PSA level (ng/mL);
- International Prostate Symptom Score (IPSS);
- Maximum urinary flow rate (Qmax, mL/s);
- Post-void residual urine volume (PVR, mL);
- Total prostate and adenoma volume (MRI, mL);
- Quality of life (QoL) score;
- International Index of Erectile Function (IIEF-5);
- Male Sexual Health Questionnaire—Ejaculatory Dysfunction (MSHQ-ED) and Bother (MSHQ-Bother) scores;
- Volume of necrosis after TPLA (MRI);
- Postoperative complications such as hematuria, dysuria, prostate abscess, and other adverse events were monitored throughout the follow-up period.

### 2.5. Statistical Analysis

Statistical analyses were performed using SPSS version 22.0 (IBM Corp., Armonk, NY, USA). The normality of data distribution was evaluated using the Kolmogorov–Smirnov test. Continuous variables were presented as mean ± standard deviation (SD) or median (interquartile range, IQR), as appropriate. Categorical variables were expressed as frequency and percentage. Repeated measures ANOVA was used for normally distributed variables to evaluate changes over time, while the Friedman test was applied for non-normally distributed variables. A *p*-value of <0.05 was considered statistically significant.

## 3. Results

### 3.1. Baseline Characteristics

A total of 53 patients were included in the study. The median age was 73 years (IQR: 18.5), and the median body mass index (BMI) was 25.7 kg/m^2^ (IQR: 3.7). At baseline, the median International Prostate Symptom Score (IPSS) was 31 (IQR: 5), with a peak urinary flow rate (Qmax) of 5.5 mL/s (IQR: 1.0) and a postvoid residual volume (PVR) of 200 mL (IQR: 62.5). The median total prostate volume was 68 mL (IQR: 20), and the adenoma volume was 36 mL (IQR: 17.5). The median quality of life (QoL) score was 4 (IQR: 1), and 75.5% of patients were using an indwelling catheter. At the time of treatment, 62.3% of patients were receiving some form of pharmacologic therapy (see Table 1). Additionally, none of the patients had a median lobe hyperplasia of the prostate demonstrated by MRI and/or TRUS, and 17 patients were using anticoagulant or antiplatelet (AC/AP) therapy for various reasons.

### 3.2. Procedural Characteristics

The TPLA procedure was completed in a median duration of 26 min (IQR: 10.5). Pain during the procedure was mild, with a median VAS score of 2 (IQR: 1). The median total energy delivered was 7200 J (IQR: 3600), and the median number of optical fibers used was 2 (IQR: 1). The median Charlson Comorbidity Index score was 3 (IQR: 3), and the majority of procedures were associated with low-grade (Clavien-Dindo grade I) or no complications (Table 2).

### 3.3. Functional and Anatomical Outcomes

Significant improvements were observed across all functional and anatomical parameters following TPLA.

- IPSSs decreased from a median of 30 at baseline to 13 at 12 months (*p* < 0.001).
- Qmax improved from 5.5 mL/s to 13.0 mL/s (*p* = 0.002).
- PVR was reduced from 200 mL to 85 mL (*p* < 0.001).
- Total prostate volume decreased from 68 mL to 40 mL (*p* = 0.020), and adenoma volume from 36 mL to 15 mL (*p* < 0.001).
- The QoL score improved significantly, from a median of 4 to 2 (*p* < 0.001).

**Figure 1:**
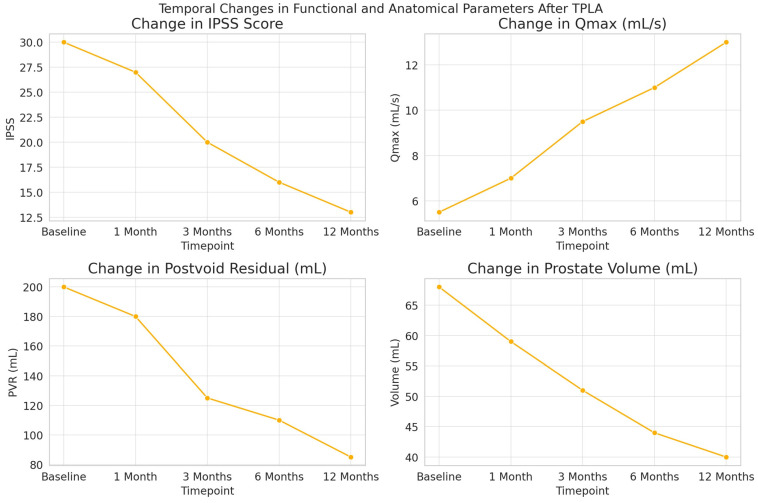
Temporal changes in functional and anatomical parameters after TPLA; Trends in functional and anatomical outcomes following transperineal laser ablation (TPLA) over 12 months. The figure illustrates progressive improvements in the International Prostate Symptom Score (IPSS), maximum urinary flow rate (Qmax), post-void residual volume (PVR), total prostate volume, and adenoma volume. Median values are plotted with interquartile ranges. IPSS and QoL scores significantly decreased, while Qmax improved and prostate parameters reduced steadily over time (all *p* < 0.05).

Additionally, prostate necrosis volume assessed by MRI increased significantly over time, from 6 mL at 1 month to 20 mL at 12 months (*p* < 0.001), indicating consistent tissue response to thermal ablation. See Figure 1 for the graphical representation of the changes in IPSS, Qmax, PVR, and prostate volume over time. Detailed numeric data are presented in Table 3. Additionally, none of our patients required repeat TPLA or conversion to surgery. The most common side effect was dysuria, which started in the first days after catheter removal and decreased over time. Importantly, no cases of hematuria, prostate abscess, orchitis, or other significant complications were observed during the entire follow-up period.

### 3.4. Sexual Function and Changes in Treatment Requirements

Overall, sexual function remained stable throughout the follow-up period. IIEF-5 scores did not show any statistically significant changes over 12 months (*p* > 0.05), indicating preserved erectile function. However, more favorable outcomes were observed in domains related to ejaculatory function. Specifically, the MSHQ-Ejaculatory Dysfunction (MSHQ-ED) score demonstrated a significant improvement by the 12th month (*p* = 0.011), suggesting enhanced ejaculatory function. In addition, MSHQ-Bother scores showed a decreasing trend over time, reaching statistical significance at 12 months (*p* = 0.013), reflecting a reduction in patients’ bother related to ejaculatory issues. (see Table 3).

Moreover, a substantial reduction was observed in the need for medical therapy. While 62.3% of patients were on pharmacological treatment prior to TPLA, this rate dropped to 35.8% by the end of the follow-up period. Similarly, a significant proportion of patients who were catheter-dependent at baseline were able to resume spontaneous voiding post-procedure (*p* = 0.001).

## 4. Discussion

The results of our retrospective study demonstrate that ultrasound-guided transperineal laser ablation (TPLA) is an effective, well-tolerated, and minimally invasive technique for the treatment of lower urinary tract symptoms (LUTS) secondary to benign prostatic hyperplasia (BPH). We observed significant and progressive improvements in IPSS, Qmax, PVR, and prostate volume over a 12-month follow-up. Additionally, ejaculatory function was preserved and even improved in some patients, aligning with prior studies reporting favorable sexual function outcomes following TPLA [10,15]. Our findings corroborate the results reported by Sessa et al. [22], who demonstrated that ultrasound-guided TPLA is a safe and effective minimally invasive option for BPH treatment with favorable functional outcomes at 12 months.

Unlike other minimally invasive surgical therapies (MISTs) such as HoLEP or photoselective vaporization, which are associated with high rates of retrograde ejaculation—70% and 63%, respectively [23]—TPLA has shown negligible ejaculatory dysfunction, due to its precise perineal approach that avoids direct urethral instrumentation and spares critical structures such as the bladder neck and ejaculatory ducts [10,24]. Similar to the observations by Sessa et al. [22], TPLA in our cohort preserved ejaculatory function, likely due to the precise perineal approach avoiding critical anatomical structures. This is particularly valuable for sexually active patients, where preservation of ejaculation is directly linked to treatment satisfaction and quality of life [23,25].

The Ultrasound and MRI-based follow-up in our study enabled accurate tracking of coagulative necrosis and prostate volume remodeling, supporting the notion that TPLA induces a gradual cytoreductive process rather than immediate mechanical debulking [26,27]. Echoing the findings of Sessa et al. [22], our MRI assessments support that TPLA induces gradual prostate volume reduction through thermal coagulation rather than immediate tissue removal. Furthermore, Manenti et al. [28] validated the use of 3-T MRI for detailed visualization and clinical monitoring of tissue changes following ultrasound-guided transperineal laser ablation, reinforcing the reliability of advanced imaging techniques in assessing treatment efficacy. Similar patterns of progressive symptomatic and anatomic improvement were described in earlier reports [15,29], suggesting that the full benefits of TPLA may become more pronounced in medium- to long-term follow-up.

In line with these observations, the recent systematic review and pooled analysis by Tafuri et al. [30] reported that TPLA shows promising safety and efficacy profiles with significant symptom improvement and prostate volume reduction up to 12 months post-procedure. However, the authors caution that the current evidence is derived mostly from small pilot studies with limited follow-up duration, underscoring the need for larger prospective trials to establish long-term durability and comparative effectiveness. Similarly, a prospective cohort study of 100 patients reported consistent functional improvements with minimal complications but emphasized the necessity of longer follow-up to confirm sustained benefits [31].

Safety outcomes in our cohort were consistent with those in previous series [15], with no major complications such as bleeding, infection, or incontinence. The most common adverse event was transient dysuria, which may reflect the post-ablative inflammatory response and can be managed effectively with perioperative NSAIDs and corticosteroids, as suggested by Pacella et al. [15]. In agreement with the safety profile described by Sessa et al. [22], our study observed no major complications, with transient dysuria being the most common manageable adverse event. Importantly, no patients in our study required reoperation or conversion to TURP, whereas progression rates to TURP have been reported at 6.5–20% for UroLift and PAE [29,32].

Functionally, the improvements in IPSS and Qmax in our cohort are comparable to those reported with TURP or Aquablation [25,33], and superior to those observed with non-ablative MISTs such as UroLift, iTIND, and PAE [15,34]. Our patients also showed significant reductions in prostate volume and adenoma size on MRI, supporting the tissue-targeted effect of thermal laser coagulation [15].

Another key advantage of TPLA is its applicability in outpatient settings and its suitability for elderly or high-risk patients who are unfit for general or spinal anesthesia [35,36]. In our study, the median procedure time was 26 min, and all cases were completed under local anesthesia. These findings support previous reports highlighting the short procedural time, minimal invasiveness, and low perioperative morbidity of TPLA [10,12,15]. Our study findings are consistent with the existing literature, highlighting the minimally invasive nature of TPLA and its suitability for outpatient procedures as well as for elderly patients with comorbidities [22].

Despite these encouraging results, several limitations should be acknowledged. First, the study’s single-arm design and relatively small sample size limit the ability to make comparative conclusions. Second, the absence of a control group prevents direct evaluation against standard therapies such as TURP or other MISTs. Similar to limitations acknowledged by Sessa et al. [22], the retrospective single-center design and lack of control group in our study constrain the generalizability of the findings. Additionally, the majority of patients in our cohort were catheter-dependent, which may further impact the generalizability of the results to the broader BPO patient population. Third, cost-effectiveness data were not analyzed in our cohort. Although the outpatient feasibility and minimal resource requirements of TPLA suggest potential economic advantages, this aspect requires formal evaluation through health economic modeling, as highlighted in other reports comparing MISTs [37,38].

While the 12-month outcomes in our study are encouraging, it is prudent to adopt a cautious interpretation regarding the long-term durability of TPLA. Current evidence, including recent systematic reviews and prospective cohort studies [30,31], primarily reflects short- to mid-term follow-up data, which may not fully capture the natural progression of BPH or late complications. Therefore, further large-scale, prospective, randomized controlled trials with extended follow-up are essential to confirm the sustained efficacy and safety of TPLA, as well as to position this modality appropriately within the spectrum of available BPH treatments.

## 5. Conclusions

TPLA appears to be a promising treatment option for LUTS due to BPH, demonstrating encouraging symptom relief, prostate volume reduction, and preservation of sexual function, with minimal morbidity over the short to medium term. When performed by experienced operators, TPLA may offer a viable alternative to traditional surgical or ablative therapies, particularly for patients who are unfit for or reluctant to undergo more invasive interventions. However, given the currently limited duration of follow-up and relatively small sample sizes in existing studies, further long-term prospective research is necessary to confirm the durability of these outcomes and to fully establish the comparative effectiveness of TPLA within the spectrum of available treatments.

## Figures and Tables

**Table 1:** Baseline sociodemographic and clinical characteristics of the patients.

Characteristic		*n* = 53
Age (years), median (IQR)		73 (18.5)
BMI (kg/m^2^), median (IQR)		25.7 (3.7)
PSA (nmol/mL), median (IQR)		7.84 (7.50)
IPSS, median (IQR)		31 (5)
Qmax (mL/s), median (IQR)		5.5 (1)
PVR (mL), median (IQR)		200 (62.5)
Prostate volume (mL), median (IQR)		68 (20)
Prostate adenoma volume (mL), median (IQR)		36 (17.5)
QoL score, median (IQR)		4 (1)
IIEF-5 score, median (IQR)		9 (7)
MSHQ-ED, median (IQR)		5 (6)
MSHQ-Bother, median (IQR)		2 (1)
Charlson Comorbidity Index, median (IQR)		3 (3)
Use of Medical Therapy	No medical treatment n(%)	20 (37.7)
	Only alpha-blocker n(%)	18 (34.0)
	Alpha-blocker + 5-alpha-reductase inhibitör n(%)	13 (24.5)
	Alpha-blocker + anticholinergic n(%)	2 (3.8)
Catheter Use Follow-up/Glob Vesicale Status	Not present n(%)	13 (24.5)
	Present n(%)	40 (75.5)

Qmax: peak flow rate; PVR: post-void residual; IPSS: International Prostatic Symptoms Score; QoL: Quality of Life; IIEF−5: International Index of Erectile Function; MSHQ-ED: Male Sexual Health Questionnaire for Ejaculatory Dysfunction; IQR: Interquartile Range.

**Table 2:** Procedural characteristics of TPLA.

Parameter	Median (IQR)
Procedure duration (minutes)	26 (10.5)
Pain during procedure (VAS score)	2 (1)
Total energy applied (Joule)	7200 (3600)
Number of probes used (n)	2 (1)
Clavien-Dindo complication grade	1 (0)

VAS: Visual Analog Scale; IQR: Interquartile Range.

**Table 3:** Functional and anatomical outcomes after TPLA.

Parameter		Pre-Treatment	Post-Treatment							
			1st Month		3rd Month		6th Month		12th Month	
				*p*		*p*		*p*		*p*
PSA (nmol/mL), median (IQR)		7.84 (7.50)	7.4 (6.55)	<0.001	5.9 (5.3)	<0.001	5 (4.9)	<0.001	4.5 (4.8)	<0.001
IPSS, median (IQR)		30 (6)	27 (10)	<0.001	20 (9)	<0.001	16 (9)	<0.001	13 (8)	<0.001
Qmax (mL/s), median (IQR)		5.5 (1.0)	7.0 (1.0)	0.020	9.5 (1.8)	0.005	11.0 (1.0)	0.003	13.0 (1.9)	0.002
PVR (mL), median (IQR)		200 (62.5)	180 (27.5)	0.001	125 (27.5)	<0.001	110 (17.5)	<0.001	85 (10)	<0.001
Prostate volume (mL), median (IQR)		68 (20)	59 (17.5)	<0.001	51 (16.5)	<0.001	44 (11)	<0.001	40 (11)	0.020
Prostate adenoma volume (mL), median (IQR)		36 (17.5)	30 (12)	<0.001	22 (13)	<0.001	19 (12)	<0.001	15 (12)	<0.001
QoL score, median (IQR)		4 (1)	4 (2)	<0.001	3 (2)	<0.001	3 (1)	<0.001	2 (0.5)	<0.001
IIEF-5 score, median (IQR)		9 (7)	8 (8)	<0.001	9 (8.5)	0.580	8 (8)	0.849	8 (9.5)	0.470
MSHQ-ED, median (IQR)		5 (6)	5 (6)	0.016	5 (6.5)	0.840	6 (6)	0.329	7 (7)	0.011
MSHQ-Bother, median (IQR)		2 (1)	2 (1)	0.853	3 (1)	0.172	3 (1)	0.053	3 (1)	0.013
Necrosis volume after TPLA (mL), median (IQR)		-	6 (2.5)	-	11 (6.5)	<0.001	16 (6)	<0.001	20 (7.5)	<0.001
Use of Medical Therapy	No medical treatment n(%)	20 (37.7)	23 (43.4)	<0.001	24 (45.3)	<0.001	31 (58.5)	<0.001	34 (64.2)	0.007
	Only alpha-blocker n(%)	17 (34.0)	14 (26.4)		13 (24.5)		9 (17.0)		9 (17.0)	
	Alpha-blocker + 5-alpha-reductase inhibitör n(%)	14 (26.4)	14 (26.4)		14 (26.4)		13 (24.5)		10 (18.9)	
	Alpha-blocker + anticholinergic n(%)	2 (3.8)	2 (3.8)		2 (3.8)		0 (0)		0 (0)	
Catheter Use Follow-up/Glob Vesicale Status	Not present n(%)	13 (24.5)	13 (24.5)	0.001	19 (35.8)	0.001	26 (49.1)	0.003	28 (52.8)	0.001
	Present n(%)	40 (75.5)	40 (75.5)		34 (64.2)		27 (50.9)		25 (47.2)	

Qmax: peak flow rate; PVR: post-void residual; IPSS: International Prostatic Symptoms Score; QoL: Quality of Life; IIEF−5: International Index of Erectile Function; MSHQ-ED: Male Sexual Health Questionnaire for Ejaculatory Dysfunction; IQR: Interquartile Range.

## Data Availability

The datasets generated and/or analyzed during the current study are not publicly available due to ethical and privacy restrictions but can be made available from the corresponding author upon reasonable request and with appropriate approval.

## Abbreviations

The following abbreviations are used in this manuscript:

BPH	Benign Prostatic Hyperplasia
LUTS	Lower Urinary Tract Symptoms
TRUS	Transrectal Ultrasonography
MRI	Magnetic Resonance Imaging
Qmax	Maximum Urinary Flow Rate
IPSS	International Prostate Symptom Score
PVR	Post-Void Residual Urine Volume
TPLA	Transperineal Laser Ablation
UDS	Urodynamic Studies
PSA	Prostate-Specific Antigen
QoL	Quality of Life
IIEF-5	International Index of Erectile Function
MSHQ-ED	Male Sexual Health Questionnaire for Ejaculatory Dysfunction
VAS	Visual Analog Scale
IQR	Interquartile Range
